# Cardiorenal benefits of finerenone: protecting kidney and heart

**DOI:** 10.1080/07853890.2023.2171110

**Published:** 2023-01-31

**Authors:** José R. González-Juanatey, Jose Luis Górriz, Alberto Ortiz, Alfonso Valle, Maria Jose Soler, Lorenzo Facila

**Affiliations:** a Cardiology Department, Hospital Clínico Universitario Santiago de Compostela, Centro de investigación Biomédica en Red Enfermedades Cardiovasculares (CIBERCV), Santiago de Compostela, Spain; b Nephrology Department, Hospital Clínico Universitario de Valencia, Universidad de Valencia, Valencia, Spain; c Nephrology Department, Fundación Jiménez Díaz, Madrid, Spain; d Cardiology Department, Hospital La Salud, Valencia, Spain; e Nephrology Department, Hospital Universitario Vall d‘Hebron, Barcelona, Spain; f Cardiology Department, Consorcio Hospital General Universitario de Valencia, Valencia, Spain

**Keywords:** Albuminuria, cardiovascular, chronic kidney disease, finerenone, inflammation, type 2 diabetes

## Abstract

Persons with diabetes and chronic kidney disease (CKD) have a high residual risk of developing cardiovascular (CV) complications despite treatment with renin-angiotensin system blockers and sodium-glucose cotransporter type 2 inhibitors. Overactivation of mineralocorticoid receptors plays a key role in the progression of renal and CV disease, mainly by promoting inflammation and fibrosis. Finerenone is a nonsteroidal selective mineralocorticoid antagonist. Recent clinical trials, such as FIDELIO-DKD and FIGARO-DKD and the combined analysis FIDELITY have demonstrated that finerenone decreases albuminuria, risk of CKD progression, and CV risk in subjects with type 2 diabetes (T2D) and CKD. As a result, finerenone should thus be considered as part of a holistic approach to kidney and CV risk in persons with T2D and CKD. In this narrative review, the impact of finerenone treatment on the CV system in persons with type 2 diabetes and CKD is analyzed from a practical point of view.

**Key messages:**
- Despite inhibition of renin-angiotensin system and sodium-glucose cotransporter type 2, persons with type 2 diabetes (T2D) and chronic kidney disease (CKD) remain on high cardiovascular (CV) residual risk.
- Overactivation of mineralocorticoid receptors plays a key role in the progression of renal and CV disease, mainly by promoting inflammation and fibrosis that is not targeted by traditional treatments.
- Finerenone is a nonsteroidal selective mineralocorticoid antagonist that decreases not only albuminuria, but also the risk of CKD progression, and CV risk in subjects with T2D and CKD.

## 1. Introduction

Diabetes is a worldwide public health problem that is set to worsen in the coming years as a result of aging and unhealthy lifestyle habits, most notably insufficient physical activity and obesity [1]. Indeed, the International Diabetes Federation projects that, based on current trends and in the absence of sufficient action to address the situation, the number of adults with diabetes will rise from 540 million in 2021 to 780 million in 2045 [2], by which time diabetes will be the seventh, and chronic kidney disease (CKD) the fifth, leading global cause of death [3]. Diabetes is one of the main causes of CKD. About 40% of persons with diabetes have stage 1 − 4 CKD and, in turn, about 40% of cases of end-stage CKD are due to diabetes [4].

Diabetes and CKD are independent risk factors for reduced survival. When both are present in the same person, the risk of mortality and of developing cardiovascular (CV) disease multiplies [5]. Individuals with CKD and diabetes are three times more likely to experience CV death than those with diabetes alone [5]. Albuminuria and decreased glomerular filtration rate are independently associated with CV mortality, risk of developing heart failure (HF), and atherosclerotic CV disease [6,7]. The European Society of Cardiology has identified albuminuria and glomerular filtration rate, along with glycemia and LDL cholesterol, as the four baseline analytical determinations to stratify cardiovascular risk and guide person management [8]. Albuminuria and glomerular filtration rate should be considered as therapeutic targets to reduce the burden of CV disease in persons with CKD [9]. In persons with stage 3 CKD, the risk of death, mainly of CV origin, is up to 10 times higher than the risk of progressing to end-stage kidney disease [10,11]. As such, strategies that protect the kidney and also reduce the risk of developing CV events should be strongly considered in individuals with diabetes and CKD [12].

Traditionally, angiotensin-converting enzyme (ACE) inhibitors and angiotensin II receptor antagonists (ARBs) have been used to reduce the risk of kidney disease progression and CV complications in individuals with kidney disease and diabetes [13–15]. Unfortunately, a substantial proportion of persons continue to present renal and CV complications despite treatment, indicating that renin-angiotensin system blockade alone is insufficient [13–15]. More recently, the addition of sodium-glucose cotransporter type 2 (SGLT2) inhibitors, in particular, canagliflozin in CREDENCE, dapagliflozin in DAPA-CKD, and empagliflozin in EMPA-KIDNEY, to renin-angiotensin system blockade has shown benefit in persons with CKD in terms of reducing kidney disease progression and the occurrence of CV events [16–18]. Despite this combination of therapeutics, an important residual risk of CKD progression and CV events remains [19,20].

**Figure 1.**
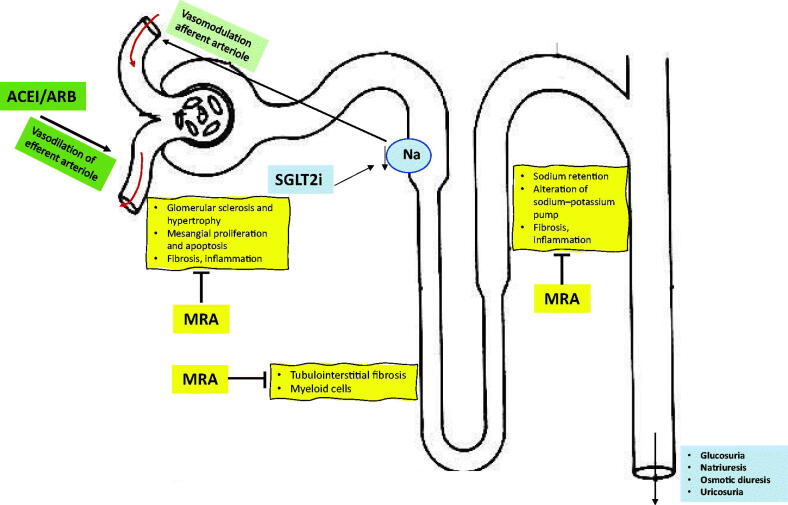
Mechanisms of action of ACE inhibitors/ARB, SGLT2 inhibitors and MRA on kidneys. ARB: angiotensin receptor blockers; ACE: angiotensin converting enzyme; SGLT2: sodium-glucose cotransporter type 2; MRA: mineralocorticoid receptor antagonists. Figure made with reference data [19,24–27].

The genesis and progression of CKD in persons with type 2 diabetes (T2D) involvings hemodynamic factors (arterial hypertension and increased intraglomerular pressure), metabolic factors (poor glycemic control), and overactivation of the mineralocorticoid receptor (MR) which promotes inflammation and fibrosis, ultimately leading to alterations at the cardiac, vascular and renal levels [20,21]. As dual therapy, renin-angiotensin system blockers and SGLT2 inhibitors act on hemodynamic factors and SGLT2 inhibitors (as well as other antihyperglycemic drugs) act on metabolic factors. Since inflammatory and fibrotic factors are not as well characterized as therapeutic targets for CKD in diabetes, this may explain the residual risk of CKD progression and CV events. A superior approach may be to use treatments that act globally to control all factors that influence the etiopathogenesis of CKD in persons with diabetes, including increased inflammation and fibrosis [19–23]. The different mechanisms of action of ACE inhibitors/ARB, SGLT2 inhibitors, and MRA are summarized in Figure 1 [19,24–27].

Finerenone is a new selective and non-steroidal MR antagonist indicated for the treatment of CKD in T2D persons [28]. The benefits of finerenone are not limited to slowing the progression of kidney disease, as it has been shown to also prevent CV complications in this population [29–31]. In this narrative review, we examined the impact of finerenone treatment on the CV system in persons with T2D and CKD from a practical point of view. For this purpose, a literature search was performed in PubMed and Embase on November 2022. We used medical evidence subject heading (MeSH) related to finerenone combined with the operator ‘AND’ with text word “diabetes” and “chronic kidney disease”. The reference lists of included studies were also screened for additional manuscripts. There were no language restrictions.

## 2. The mineralocorticoid receptor (MR) in the development of kidney and CV disease in individuals with T2D and CKD

In individuals with T2D and CKD there is an overarousal of the MR. The MR is expressed in numerous body cells including, among others, the distal and connecting convoluted tubule, macula densa, podocytes, mesangial cells, cardiomyocytes, vascular smooth muscle cells, endothelial cells, adipocytes, fibroblasts, and macrophages [21,22].

Through various mechanisms, overarousal of the MR increases inflammation and fibrosis, which translates into alterations at the renal level: alteration of sodium-potassium ATPase in the distal convoluted tubule and sodium retention, increased blood pressure, glomerulosclerosis and glomerular hypertrophy, mesangial proliferation, tubulointerstitial fibrosis; vascular level: endothelial dysfunction, increased arterial stiffness; and cardiac level: myocardial hypertrophy and fibrosis, ventricular remodelling, decreased coronary flow, myocardial ischemia, impaired cardiac function, arrhythmias [21–23].

**Figure 2.**
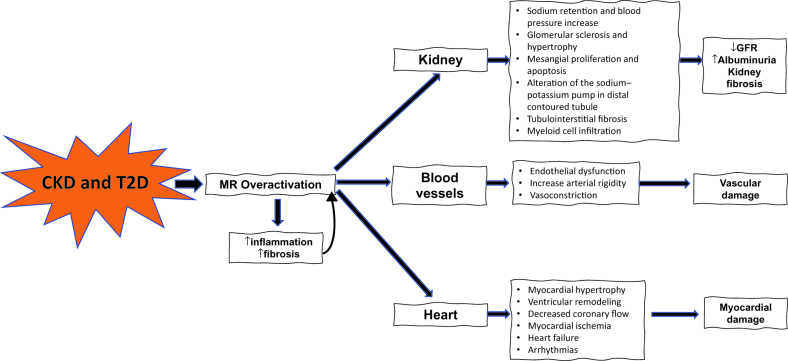
Consequences of hyperactivation of the mineralocorticoid receptor in persons with chronic kidney disease and diabetes. MR: mineralocorticoid receptor; T2D: type 2 diabetes. Figure was made with reference data [4,19–22,32,33].

Ultimately, MR overarousal contributes to the progression of CKD (decreased glomerular filtration rate and increased albuminuria), vascular damage, and CV complications (atherosclerotic coronary disease, HF, arrhythmias) (Figure 2) [4,19–22,32,33].

MR blockage produces coronary vasodilatation, increases nitric oxide, overactivation of mitochondrial pathways, and provides better perfusion of the left ventricle at the cardiac level. In addition, it improves endothelial function, decreases arterial stiffness, and reduces neointimal formation after vascular damage at the vascular level. Furthermore, MR blockage decreases albuminuria and enhances the preservation of renal function at the renal level [19,34–36].

In this manner, MR overactivation plays a central role in the development of CV and renal disease.

## 3. MR antagonists

MR antagonists (MRA) available to date are the steroid molecules, spironolactone and eplerenone, whose impact on renal progression or the development of CV complications has not been studied in persons with CKD and T2D despite their reducing proteinuria in the short term. Differences in the mechanisms of action of these drugs underlie differences in their efficacy and safety profiles which have facilitated a successful completion of phase 3 clinical trials with finerenone alone (Table 1) [4,20,28,37–39]. Classically, angiotensin was considered to be the main factor stimulating aldosterone secretion, and aldosterone was the main ligand for the MR. Aldosterone increases blood pressure by promoting sodium retention and volume expansion, and ARBs can prevent this activity. However, the role of the MR is much broader and more complex. Multiple factors trigger an over-activation of the MR, including the ligands aldosterone, cortisol, and progesterone, and the non-ligands RAC1, hyperglycemia, and excess sodium. Thus, aldosterone is one of several ligands at the MR. Overactivation of the MR increases inflammation and fibrosis, thus promoting the progression of CKD and CV disease [21,32].

**Table 1.** Characteristics of mineralocorticoid receptor antagonists.

	Steroidal MRA		Non-steroidal MRA
	Spironolactone	Eplerenone	Finerenone
Active metabolites	Yes	No	No
Half-life	≥24 h	4-6 h	2.8 h
Distribution (kidney-heart)	>6:1	≈3:1	1:1
Antagonism over MR			
Power	High	Low	High
Selectivity	Low	Medium	High (Bulky [large] and passive)
Effect on recruitment of cofactors (without aldosterone)	Partial agonist	Partial agonist	Inverse agonist
Effect on recruitment of cofactors (with aldosterone)	Inhibition	Inhibition	Blocking
Effect on inflammation and fibrosis	Moderate	Moderate	High
Blood pressure reduction	Marked	Marked	Moderate (greater at high doses)
Effect on proteinuria and kidney damage	Moderate	Moderate	Powerful
Hyperkalemia risk	High	High	Moderate
Other adverse effects	Gynecomastia		

Table created with reference data [4,20,28,37–39]. MR: mineralocorticoid receptor; MRA: mineralocorticoid receptor antagonists.

There are notable differences between steroidal MRAs (spironolactone and eplerenone) and non-steroidal MRAs (finerenone), depending on their structure (Table 1) which is quasi-flat for steroidal MRAs and voluminous in three dimensions for finerenone. Finerenone is selective for the MR and its binding to the receptor gives rise to a complex that blocks the recruitment of transcriptional coactivators involved in the expression of proinflammatory and profibrotic mediators [37–39]. However, steroidal MRAs act as partial agonists on cofactor recruitment in the absence of aldosterone [37–39]. In fact, they activate the MR in individuals with the genetic variant S810L, causing early severe hypertension [40]. This alters the effect on proteinuria and kidney damage, with finerenone having a greater effect than spironolactone and eplerenone [38,39]. Furthermore, in pre-clinical studies, finerenone was shown to have a homogeneous distribution between the kidney and heart, while spironolactone and eplerenone are preferentially concentrated in the kidney, which increases the risk of hyperkalemia (Table 1) [4,20,28,37–39].

Finerenone is currently approved for the treatment of CKD (stage 3 and 4 with albuminuria) associated with T2D [28], since it reduces the risk of the two main complications of CKD, CKD progression and high risk of CV disease and death [29–31]. Finerenone has rapid absorption and elimination. As it is metabolized *via* CYP3A4, it should not be co-administered with potent inhibitors or inducers of CYP3A4. The recommended target dose of finerenone is 20 mg once daily. The starting dose (10 mg or 20 mg od) depends on the glomerular filtration rate, and dose titration (10 mg or 20 mg od) on the serum potassium levels. It is recommended that renal function and serum potassium are measured at baseline, and 4 weeks after any change in dose of finerenone (Table 2) [28].

**Table 2.** Practical aspects of finerenone prescription.

Indication	Adults with CKD (stages 3 and 4 with albuminuria) associated with type 2 diabetes
Time to maximum concentration	0.5–1.0 h
Terminal half-life	≈2–3 h
Target dose	20 mg once a day
Maximum recommended dose	20 mg once a day
Starting dose	eGFR ≥60 mL/min/1.73 m^2^: 20 mg/day eGFR ≥25–60 mL/min/1.73 m^2^: 10 mg/day eGFR <25 mL/min/1.73m^2^: not recommended
Maintenance dose according to serum potassium	*K* ≤ 4.8 mmol/L: If 10 mg increase to 20 mg (except if eGFR ↑ >30%); if 20 mg, maintain dose *K* > 4.8–5.5 mmol/L: maintain dose (10 or 20 mg) *K* > 5.5 mmol/L: interrupt treatment and consider reinitiation if ≤ 5.0 mmol/L (with 10 mg)
Monitoring	Measure K and eGFR at baseline and at 4 weeks (after initiation, restart, or dose increase)
Dose adjustment	Age, weight, mild to moderate hepatic impairment: not necessary Severe hepatic impairment: use not recommended (lack of clinical data)
Pharmacological interactions	Strong CYP3A4 inducers or inhibitors; do not use Moderate or weak CYP3A4 inhibitors: can be taken (closer monitoring)

CKD: chronic kidney disease; eGFR: estimated glomerular filtration rate; K:serum potassium. Table made with reference data [28].

## 4. Finerenone slows the progression of CKD and reduces the risk of CV events

Multiple preclinical and clinical studies have shown the renal and CV benefits of finerenone, as well as its safety.

In ovariectomized mice with left ventricular diastolic dysfunction and preserved ejection fraction, finerenone improved left ventricular filling, coronary artery endothelium-dependent relaxation, mitochondrial ATP production, and exercise capacity [41]. Experimental studies of finerenone have also shown improvement in metabolic parameters, such as the average size of adipocytes, adipocyte differentiation, fat deposits, type of fat, and oxidative stress [42,43].

Phase II clinical trials of finerenone were ARTS (Mineralocorticoid Receptor Antagonist Tolerability Study), ARTS-HF (MinerAlocorticoid Receptor Antagonist Tolerability Study-Heart Failure), and ARTS-DN (ARTS-Diabetic Nephropathy) [44–46]. In part B of ARTS, finerenone (2.5 − 10 mg/day) was compared with placebo and open-label spironolactone (25 or 50 mg/day) in 392 persons with HF with reduced ejection fraction and moderate CKD. Finerenone reduced natriuretic peptides and albuminuria to a similar extent than spironolactone. However, the risk of hyperkalemia was lower with finerenone than with spironolactone (5.3 vs 12.7%; *p* = 0.048) [44]. In ARTS-HF, which randomized 1066 persons with HF and reduced ejection fraction and CKD and/or diabetes, finerenone reduced natriuretic peptides similarly to eplerenone, and was associated with a trend towards fewer events (death from any cause, CV hospitalization, or emergency room visits for worsening HF up to day 90), which reached statistical significance at day 90 in the finerenone 10 to 20 mg group (hazard ratio [HR] 0.56; 95% confidence interval [CI] 0.35–0.90; nominal *p* = 0.02) [45]. ARTS-DN randomized 823 persons with T2D and albuminuria (urinary albumin-creatinine ratio >30 mg/g) under treatment with ACE inhibitors or ARBs. Finerenone (7.5, 10, 15, and 20 mg orally daily) significantly reduced albuminuria compared with placebo at 90 days of treatment (HR 0.79; 90% CI 0.68–0.91; *p* = 0.004, HR 0.76; 90% CI 0.65–0.88; *p* = 0.001, HR 0.67; 90% CI 0.58–0.77; *p* < 0.001, and HR 0.62; 90% CI 0.54–0.72; *p* < 0.001, respectively). In addition, hyperkalemia leading to discontinuation was reported in 2.1%, 0%, 3.2%, and 1.7%, of finerenone groups, respectively (vs 0% in the placebo group) [46].

In a systematic review that analyzed three clinical trials involving 1520 persons in total with HF and reduced ejection fraction, finerenone was found to have lowered natriuretic peptides similarly as steroid MRAs, but with a lower risk of hyperkalemia and higher glomerular filtration rate [47]. In another meta-analysis of 13 clinical trials involving 13,597 persons with HF and reduced ejection fraction, finerenone 10 mg reduced the risk of CV death, hospitalization, and adverse events compared to spironolactone and eplerenone [48].

Inarguably, the best understanding of the benefits of finerenone in persons with T2D and CKD has derived from the pivotal phase 3 clinical trials: FIDELIO-DKD (Finerenone in Reducing Kidney Failure and Disease Progression in Diabetic Kidney Disease) [29]; FIGARO-DKD (Finerenone in Reducing Cardiovascular Mortality and Morbidity in Diabetic Kidney Disease) [30]; and the pre-specified pooled analysis FIDELITY (Combined FIDELIO-DKD and FIGARO-DKD Trial program analysis) [31].

**Figure 3.**
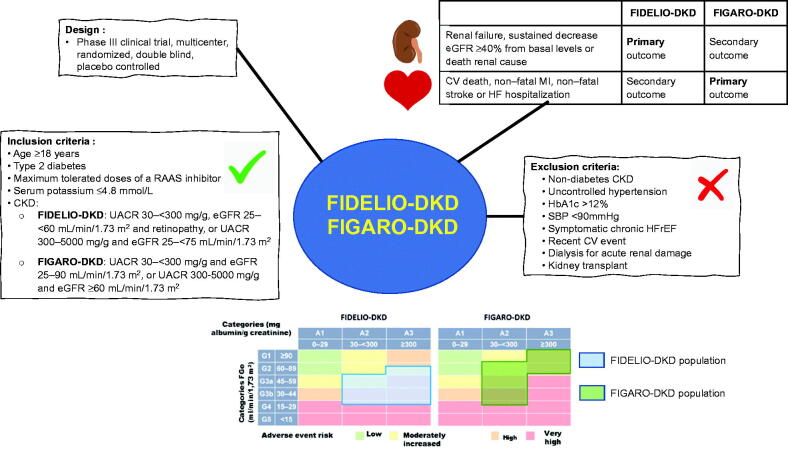
General characteristics of the FIDELIO-DKD and FIGARO-DKD studies. UACR: urine albumin-creatinine ratio; CV: cardiovascular; CKD: chronic kidney disease; eGFR: estimated glomerular filtration rate; HFrEF: heart failure with reduced ejection fraction; MI: myocardial infarction; SBP: systolic blood pressure. Figure made with data from the references [29,30].

FIDELIO-DKD included approximately 5700 persons with T2DM and CKD who were receiving maximum tolerated doses of an ACE inhibitor or ARB and had a serum potassium concentration ≤ of 4.8 mmol/L (Figure 3) [29]. After a mean follow-up of 2.6 years, relative to placebo, finerenone significantly reduced CKD progression (primary endpoint) by 18% (HR 0.82; 95% CI = 0.7–0.93; *p* = 0.001], and decreased the composite secondary endpoint (death from CV causes, non-fatal myocardial infarction, nonfatal stroke, or hospitalization for HF) by 14% (HR 0.86; 95% CI = 0.75–0.99) (Table 3). The benefit of finerenone was independent of the history of CV disease [11], blood pressure [49] and HbA1c [50] levels, as well as the use of insulin [50], SGLT2 inhibitors [51] or glucagon-like peptide receptor type 1 (GLP1) agonists [52]. In a secondary analysis of the FIDELIO-DKD trial, finerenone reduced the risk of newly diagnosed atrial fibrillation/flutter compared with placebo (3.2% vs 4.5%; *p* = 0.016) [53]. Although the incidence of severe hyperkalemia was higher with finerenone than placebo (1.6% vs 0.4%), the risk of serious adverse effects requiring discontinuation was similar in both treatment groups. Finerenone slightly reduced systolic blood pressure at 12 months (2.1 mmHg vs. 0.9 mmHg increase with placebo) [29].

**Table 3.** Main results of the FIDELIO-DKD, FIGARO-DKD studies and the combined FIDELITY analysis.

	FIDELIO-DKD	FIGARO-DKD	FIDELITY
	HR (95% CI)	HR (95% CI)	HR (95% CI)
Composite CV primary variable^a^	0.86 (0.75–0.99)	0.87 (0.76–0.98)	0.86 (0.78–0.95)
CV death	0.86 (0.68–1.08)	0.90 (0.74–1.09)	0.88 (0.76–1.02)
Myocardial infarction non-fatal	0.80 (0.58–1.09)	0.99 (0.76–1.31)	0.91 (0.74–1.12)
Non-fatal stroke	1.03 (0.76–1.38)	0.97 (0.74–1.26)	0.99 (0.82–1.21)
Heart failure hospitalization	0.86 (0.68–1.08)	0.71 (0.56–0.90)	0.78 (0.66–0.92)

CI: 95% confidence interval; CV: cardiovascular; HR: Hazard ratio. Table was prepared with data from references [29–31].

^a^Time to first CV death, nonfatal myocardial infarction, non-fatal stroke, or heart failure hospitalization.

The FIGARO-DKD trial [30] included more than 7000 persons with T2D and CKD who were receiving maximum tolerated doses of an ACE inhibitor or ARB and had a serum potassium concentration ≤4.8 mmol/L (Figure 3). After a mean follow-up of 3.4 years, there was a significant 13% decrease in the composite primary outcome of death from CV causes, non-fatal myocardial infarction, nonfatal stroke, or hospitalization for HF (HR 0.87; 95% CI = 0.76–0.98), as well as a trend towards less CKD progression (secondary outcome) with finerenone compared to placebo. Finerenone was associated with a significantly reduction in incident HF as indicated by numerous secondary endpoints. Finerenone significantly reduced the risk of HF hospitalizations by 29% (HR 0.71; 95% CI = 0.56–0.90) (Table 3), the risk of new cases of HF by 32% (HR 0.68; 95% CI 0. 50-0.93), the risk of CV death or first hospitalization for HF by 18% (HR 0.82; 95% CI = 0.70–0.95), the risk of first hospitalization for HF by 29% (HR 0.71; 95% CI = 0.56-0.90), and the risk of total hospitalizations for HF by 30% (HR 0.70; 95% CI = 0.52–0.94). The benefit of finerenone on HF events was independent of HF history [54]. In FIGARO-DKD, the risk of serious adverse events requiring discontinuation was similar in both treatment groups. Although the incidence of severe hyperkalemia was slightly higher with finerenone than with placebo (0.7% vs 0.1%), no fatal hyperkalemia events occurred. Finerenone had a modest effect on systolic blood pressure [30].

**Figure 4.**
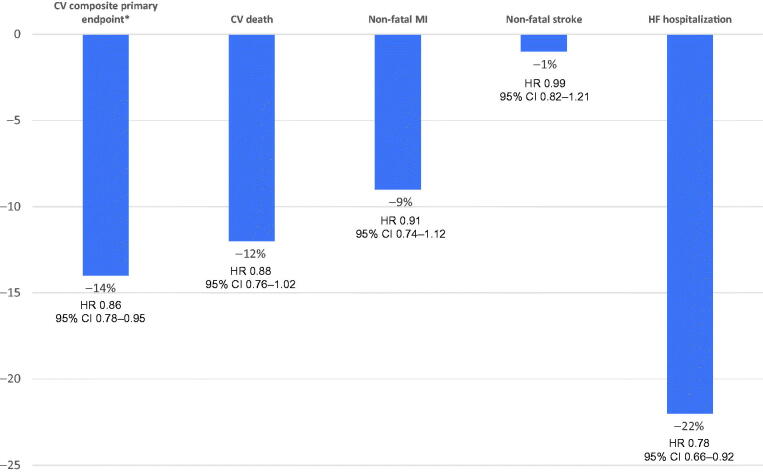
Relative risk reduction of main cardiovascular outcomes (finerenone vs. placebo) of the combined analysis of FIDELITY study. *Time to first CV death, nonfatal MI, non-fatal stroke, or HF hospitalization. CV: cardiovascular; HF: heart failure; MI: myocardial infarction; HR: Hazard ratio; 95% CI: 95% confidence interval. Figure made with reference data [31].

FIDELITY [31] was a prespecified pooled analysis of the FIDELIO-DKD and FIGARO-DKD studies. The mean age of persons was 65 years, 70% were men, 46% had a history of CV disease, and 8% had HF. According to the KDIGO risk categories associated with CKD severity [9], 48% had a very high risk and 41% had a high risk. All persons were treated with renin-angiotensin system blockade, 7.2% with GLP1 receptor agonists and 6.7% with SGLT2 inhibitors. Finerenone significantly reduced the risk of the renal composite outcome (kidney failure, sustained ≥ 57% decrease in estimated glomerular filtration rate from baseline over ≥ 4 weeks, or renal death) by 23%, the primary composite CV outcome by 14% (HR 0.86; 95% CI = 0.78–0.95) and hospitalization for HF by 22% (HR 0.78; 95% CI = 0.66–0.92) (Table 3 and Figure 4). Although the incidence of hyperkalemia was higher with finerenone, the risk of adverse effects requiring discontinuation and the incidence of acute kidney injury were similar in both treatment arms (Table 4). Another combined analysis of four clinical trials involving 7048 persons also found a reduction in CV risk with finerenone in persons with CKD, the majority of whom had T2D [55].

**Table 4.** Adverse effects in persons included in the FIDELITY analysis.

	Finerenone (*n* = 6510)	Placebo (*n* = 6489)
Adverse events		
Adverse events leading to discontinuation	6.4%	5.4%
Serious adverse events leading to discontinuation	2.2%	2.4%
Hyperkalemia		
Hyperkalemia	14.0%	6.9%
Treatment-related hyperkalemia	8.8%	3.8%
Severe hyperkalemia	1.1%	0.2%
Acute kidney injury		
Acute kidney injury	3.4%	3.6%
Hospitalization due to acute kidney injury	1.3%	1.3%
Discontinuation due to acute kidney injury	0.2%	0.2%

Table made with data from reference [31].

## 5. Integration of finerenone into the global management of CV and renal risk in persons with T2D and CKD

Individuals with T2D and CKD are at high risk of progressing to end-stage kidney disease, and even more so of developing CV complications, including atherosclerotic coronary artery disease and HF, or of dying prematurely, especially if there is prior CV disease [3–5]. In this sense, the presence of diabetes and CKD is common in persons with ischemic heart disease and those with HF [56–58]. On the other hand, it is important to note that the presence of albuminuria itself increases the risk of renal complications, CV, and death in the general population and in individuals with T2D [7]. For this reason, it is essential to determine albuminuria in persons with CKD and/or T2D, a population that is frequent in cardiology consultations, to better stratify CV risk and optimize CV protective treatment [8,56–58].

**Figure 5.**
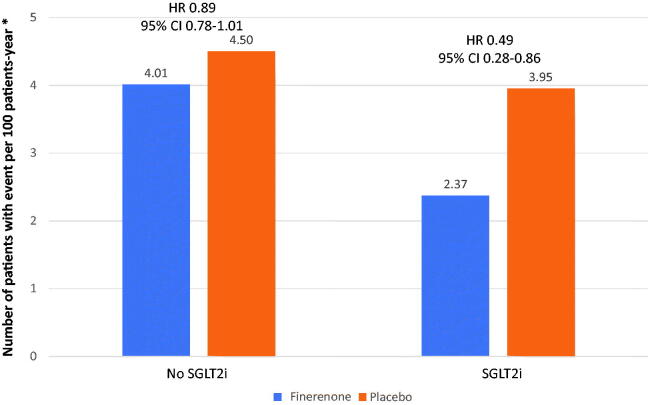
Primary cardiovascular endpoint* in the FIGARO-DKD study, depending on baseline treatment with SGLT2 inhibitors. *Death from cardiovascular causes, non-fatal myocardial infarction, nonfatal stroke, or hospitalization for heart failure. CI: confidence interval; HR: Hazard ratio; SGLT2i: SGLT2 inhibitors. Figure made with reference data [30].

Renin-angiotensin system blockers (ACE inhibitors or ARBs) and SGLT2 inhibitors have been shown to slow the progression of CKD and reduce CV risk in individuals with diabetes and CKD [13–18], but the significant residual risk remains. In this context, finerenone is an effective and safe treatment. In FIDELIO-DKD and FIGARO-DKD, all persons were under treatment with ACE inhibitors or ARBs. As such, it is clear that the benefits of finerenone were additional to renin-angiotensin system blockade. Although the CONFIDENCE study (NCT NCT05254002), which is investigating the combination of empagliflozin with finerenone in subjects with CKD and T2D, is currently underway, to date no clinical trial has specifically analyzed the effect of combining an SGLT2 inhibitor with finerenone. In the combined FIDELITY analysis, less than 7% of the population was receiving SGLT2 inhibitors at baseline. However, in the subgroup analysis of the FIDELIO-DKD study, the benefits of finerenone were found to be independent of treatment with SGLT2 inhibitors [51]. In FIGARO-DKD, the SGLT2 inhibitors-finerenone group had the lowest incidence of CV events (Figure 5) [30]. Clinical data are consistent with preclinical studies. In a hypertensive rat model, the combination of finerenone with empagliflozin reduced renal and cardiac histological lesions relative to placebo-treated rats [59]. Since finerenone and SGLT2 inhibitors have complementary mechanisms of action, an added benefit of joint treatment with both drugs would be expected [4,19,32,60]. Concomitant treatment of SGLT2 inhibitors with MRA also improves safety by reducing the risk of hyperkalemia [61].

The use of finerenone in the treatment of persons with CKD and T2D is expected to be cost-effective by reducing the health system burden of CV and renal events. For example, hospitalizations for HF represent one of the most important components of healthcare costs and a year of dialysis costs €45,000 (US$47,560). For that reason, finerenone is expected to contribute to a reduction in the overall cost associated with CKD [62,63].

Recommendations about the use of finerenone have been included in 2022 and 2023 guidelines. Thus, the 2023 American Diabetes Association (ADA) guideline recommends the use of finerenone for individuals with T2D and CKD with albuminuria treated with the maximum tolerated doses of ACE inhibitors/ARB, to improve cardiovascular outcomes and reduce the risk of CKD progression (level of recommendation A) [64]. The 2022 American Association of Clinical Endocrinology (AACE) guidelines [65] and the 2022 ADA and Kidney Disease: Improving Global Outcomes (KDIGO) joint consensus [66] recommend the use of finerenone in persons with T2D, an estimated glomerular filtration rate ≥25 mL/min/1.73 m^2^, normal serum potassium concentration, and albuminuria (urine albumin-creatinine ratio ≥30 mg/g) despite a maximum tolerated dose of a renin-angiotensin system blocker (grade A).

## 6. Discussion

Persons with T2D and CKD are at high risk of developing CV events [5]. Clinical trials have demonstrated that treatment with ACE inhibitors or ARB and more recently with SGLT2 inhibitors markedly reduces the risk of renal disease progression and CV complications in this population [13–18]. ACE inhibitors/ARB and SGLT2 inhibitors act on hemodynamic factors, such as arterial hypertension and also SGLT2 inhibitors improve glycemic control. These are key components in the pathogenesis of nephropathy and diabetes [4]. However, other factors, including inflammation and fibrosis secondary to MR overactivation play a key role in the development of CKD and diabetes, but these drugs do not act on these factors [19–21]. This could explain the residual risk that remains despite treatment with ACE inhibitors/ARBs and SGLT2 inhibitors among persons with T2D and CKD [19,20].

As a result, treatment with MR antagonists could be beneficial in this clinical setting. However, not all MR antagonists have demonstrated the same benefits or are equally safe [4,20,28,37–39]. Thus, in contrast to spironolactone and eplerenone, finerenone has demonstrated to slow the progression of kidney disease and also prevent CV complications in persons with CKD and T2D on top of renin-angiotensin system inhibition [29–31]. Another important point to be resolved is whether concomitant treatment with SGLT2 inhibitors and finerenone has synergistic effects. They have complementary mechanisms of action and available data suggest that these persons could benefit from treatment with both drugs, but more data are warranted [19,30,32,51,59,60]. In addition, SGLT2 inhibitors could reduce the risk of hyperkalemia with finerenone [61].

The evidence suggests that treatment of persons with T2D and CKD should consist of triple therapy with renin-angiotensin system blockade (ACE inhibitors or ARBs), SGLT2 inhibitors and finerenone, to slow the progression of CKD and reduce the risk of developing CV complications. For this reason, it is imperative that persons most likely to benefit from triple therapy are identified. Greater dissemination of clinical trial results and CV risk prevention guidelines and more widespread implementation of the assessment of albuminuria in persons with T2D and/or CKD should be performed. Although albuminuria is routinely assessed by nephrologists, other specialities, such as primary care, cardiology and endocrinology should also incorporate albuminuria measurement into their routine practice in the management of persons with diabetes or CKD. In addition, better coordination between the different care levels would be desirable. In addition, cardiologists are used to prescribing MRA. The better safety profile of finerenone and its lower risk of hyperkalemia should facilitate MRA prescription.

However, it should be emphasized that as FIDELIO-DKD and FIGARO-DKD included individuals with T2D and CKD with estimated glomerular filtration rate ≥25 mL/min/1.73 m^2^, normal serum potassium concentration, and albuminuria, the current indication of finerenone cannot be extended to the whole population with T2D and CKD. Therefore, new studies are warranted to clarify this point. Ongoing studies are addressing the role of triple therapy (RAS blockade, SGLT2 inhibition, finerenone) in individuals with T2D and CKD (CONFIDENCE study, NCT05254002) and the efficacy and safety of finerenone in subjects with CKD without diabetes (FIND-CKD study, NCT05047263).

## 7. Conclusions

Despite current treatments, individuals with CKD and T2D continue to have a significant CV risk. Albuminuria is a key and independent risk factor for CV and renal events. Determining albuminuria, together with the measurement of glomerular filtration rate, blood glucose, and LDL cholesterol, is the first step in risk stratification that allows for the design of a prevention strategy. Determination of albuminuria is part of the clinical practice of cardiology. Finerenone is a potent and selective non-steroidal MRA that has been shown to slow the progression of CKD and reduce the risk of developing CV complications in persons with T2D and CKD. As such, finerenone should be considered as one of the mainstays of treatment for persons with T2D and CKD, along with ACE inhibitors or ARBs, and SGLT2 inhibitors.

## Data Availability

The data that support the findings of this article are available from the corresponding author upon reasonable request.
